# Cross-Sectional Examination of Hospital Visits in the Year Prior to Suicide Death in Illinois

**DOI:** 10.5811/westjem.49106

**Published:** 2026-03-02

**Authors:** Maryann Mason, Yingxuan Liu, Krina Patel, Kunal Kanwar, Ursula Alexander, Alexander Lundberg

**Affiliations:** * Northwestern University Feinberg School of Medicine, Department of Emergency Medicine, Chicago, Illinois; † Northwestern University, Buehler Center for Health Policy and Economics, Chicago, Illinois; ‡ Nova Southeastern University College of Allopathic Medicine, Davie, Florida

## Abstract

**Introduction:**

Suicide is a growing public health issue in the United States. Healthcare visits in the year prior to suicide death, including those to emergency departments (ED) and inpatient settings, may be missed opportunities for risk-screening and intervention delivery. Our objective in this study was to evaluate the distribution of hospital visits of suicide decedents in the year prior to death by setting (ED and inpatient), last visit proximity to death, and presence of suicide risk factors, and to consider each setting’s potential for reaching those at risk of suicide.

**Methods:**

Using linked data from the Illinois Hospital Discharge Data Set and the Illinois Violent Death Reporting System, we examined suicide decedent hospital visits 365 days prior to suicide death. We described the distribution of visits by setting (ED vs inpatient), timing of the last visit prior to death, and groupings of visit primary diagnosis codes, as per the *International Classification of Diseases, 10^th^ Revision*, reflecting suicide risk (deliberate self-harm, suicidal ideation, mental health disorders, and substance use disorder). The study was conducted between 2022–2025.

**Results:**

Of the 2,562 suicide decedents, 960 (37.4%) had a visit in the year preceding their death. The 960 decedents had a total of 3,131 visits, an average of 3.3. per person. Of those visits, 2,002 (63.9%) were to the ED. However, there was a greater proportion of last visits to an inpatient unit (687, 60.9%) that occurred under 180 days of death compared to last ED visits (1,060, 52.1%), *P* < .05). Inpatient visits also had higher percentages of visits for each of the suicide risk-diagnosis code groups compared to ED visits; deliberate self-harm, 22.2% (n = 251) vs 6.8% (n = 136); suicidal ideation 29% (n = 327) vs 8.6% (n = 173); mental health disorders, 5.7% (n = 64) vs 3.1% (n = 62); and substance use disorder, 75.1% (n = 848) vs 35.3% (n = 706), *P* < .05. Among both inpatient and ED visits, substance use was the most prevalent of the primary diagnosis suicide risk-factor groups endorsed, although inpatient visits had a statistically significant higher proportion of primary diagnosis codes for substance use than ED visits, 75.1% (n = 848) and 35.3% (n = 706), respectively, all *P* < .05.

**Conclusion:**

We found the proportion of suicide decedents with a hospital visit in the year prior to death was lower than other studies found for primary care settings. However, this does not mean that broad-based suicide screening and interventions would not be of value in hospital settings.7,14 Inpatient visits were fewer in number but a greater proportion of visits in closer proximity to suicide death and with suicide risk factors. This suggests that EDs may be better suited to broad-based screening and inpatient settings to targeted intervention efforts. Inpatient visits involving primary diagnosis suicide-risk factors may offer more easily identifiable opportunities for suicide prevention compared to those in ED settings, based on prevalence and temporal and logistical factors. Future interventions could consider how to systemically integrate risk screenings in both settings, particularly for patients with a diagnosis of substance use disorder.

## INTRODUCTION

Suicide is a significant and growing public health problem in the United States.^1^ In Illinois, suicide rates have been increasing since 2018, and suicide deaths now outnumber homicide deaths.^2^ The prevention of suicide is a public health priority, and progress is needed to target scarce resources to reach those most at risk. The US Centers for Disease Control and Prevention (CDC) describe suicide risk and protective factors at the individual, relationship, community, and societal levels, noting that in most cases there is no singular cause of suicide.^1^

Suicidal ideation, self-harm, presence of a mental health disorder, and substance use are among the known risk factors at the individual level. These are also conditions for which a patient may present for treatment by a clinician. Most suicide decedents have healthcare encounters (hospital visits, primary, and specialty care) in the year prior to their death. A large national study found that nearly half (49.9%) of decedents had a primary care clinician contact in the month prior to their death.^2^ An earlier systematic review found very similar rates, with 45% of suicide decedents having visited a primary care clinician in the month prior to their death.^3^

A separate study conducted in Utah estimated that 39% of suicide decedents had visited an emergency department (ED) in the year prior to their death.^4^ With the exception of the study from Utah,^4^ most studies do not address the reasons for these visits prior to death by suicide. Emergency departments in particular have a high volume of mental health crisis visits and may be a place where at-risk persons can be reached.^4^–^7^ In hospital settings, suicide risk screening can help identify those in need of support and services to mitigate suicidal behaviors. Healthcare visits prior to suicide death represent missed opportunities for intervention and ultimately suicide prevention. However, resources including clinician time are scarce, and details about these missed opportunity visits could inform more efficient deployment of suicide prevention resources.

Our objective in this study was to evaluate the distribution of ED and inpatient hospital visits of suicide decedents in the year prior to their death. We considered the number of visits by setting, last visit proximity to death, and presence of suicide risk factors as noted in the visit primary diagnosis to weigh each setting’s potential for reaching those at risk of suicide. We hypothesized that the ED setting would have more missed opportunity visits, more last visits in closer proximity to death, and more visits with suicide risk factors present than visits in inpatient settings. Findings can help guide resource allocation and increase intervention reach to those at risk.

## METHODS

### Study Design

This was a retrospective cross-sectional study of hospital visits in the year prior to death for suicide decedents in Illinois. The Northwestern University Institutional Review Board designated this project as non-human subjects research (study number STU00216939). The study follows Strengthening the Reporting of Observational Studies in Epidemiology guidelines for cross-sectional studies.

### Data Sources

Hospital visit data come from the Illinois Hospital Discharge Data (IHDD). These data are entered by hospital staff into an administrative dataset on site at the hospital setting. Data entry processes are standardized across sites. Most states maintain hospital discharge datasets, and they are commonly used in research because they contain useful core variables about hospital visits.^5^ The IHDD variables used in this study include admission/discharge dates, visit type (ED and inpatient), admit source, hospital name, and primary diagnosis based on the *International Classification of Diseases, 10^th^ Revision*, (*ICD-10*) codes. Visits to an ED are defined as a visit to an ED that does not result in hospital admission or stay. Inpatient visits are defined as an inpatient stay in an inpatient hospital unit ≥ 24 hours for a medical reason. Primary diagnosis codes are the conditions established after examination that are responsible for the admission for hospital care. Each visit can contain up to 25 primary diagnosis codes, although most have fewer. Due to data-sharing constraints, we were able to obtain only IHDD for cases that matched with a suicide death. See below for a description of the data linkage process. We determined whether a hospital was a psychiatric facility or other type by checking hospital names against the IDPH hospital directory and coding “1” for psychiatric and “0” for all other types.

**Population Health Research Capsule**

What do we already know about this issue?

*Suicide is a complex issue and difficult to prevent. Healthcare visits prior to suicide may be missed opportunities for prevention*.

What was the research question?

*Do emergency department (ED) or inpatient settings offer better opportunities for suicide risk screening and intervention?*

What was the major finding of the study?

*A greater share of inpatient visits occurred within 30 days of death (13.3% vs 9.3%) and involved suicide risk factors (85.7% vs 41.7%) compared to ED visits (P < .005)*.

How does this improve population health?

*Findings suggest that inpatient settings may offer slight advantages over ED settings to convert missed-opportunity visits to suicide intervention opportunities*.

Suicide decedent data come from the Illinois Violent Death Reporting System (IVDRS). The IVDRS, which is part of the CDC’s National Violent Death Reporting System, is a state-based public health surveillance system recording data on homicide, suicide, legal intervention, unintentional firearm, and undetermined deaths. Data for IVDRS come from death certificates and coroner/medical examiner, law enforcement, autopsy, and toxicology reports. Data abstractors review original reports and enter the data into the CDC’s online system using standardized procedures and coding guidance. For analyses we used the following IVDRS variables: date of death; sex; and race/ethnicity. Sex and race/ethnicity are determined by the coroner/medical examiner at the time of the death investigation based on physical examination and next-of-kin interviews.

### Data Linkage

To capture hospital visits in the year prior to death we used IHDD data from 2017–2018 and IVDRS data from 2018–2019. We followed a five-step process to link IHDD and IVDRS datasets. Step 1 entailed merging the CDC-certified IVDRS dataset with a master death certificate file using incident number, victim number, and incident year as keys. This process created a dataset with state file numbers, which were needed to match with the IHDD. In Step 2 we sent a limited IVDRS dataset including date of birth, sex, residence ZIP code, first and last name, state file number, last four digits of the death certificate number, and last four digits of the Social Security number to the Illinois Department of Public Health (IDPH) to conduct the IHDD linkage. In Step 3, the IDPH matched IVDRS records to hospital IHDD using the limited IVDRS dataset and returned a linked dataset including state file number, last four digits of the death certificate number, sex, admission date, discharge date, admit source, primary diagnosis codes 1–25, and hospital name variables. We then performed a final merger linking the full set of IVDRS variables to each hospital visit record, using state file number as the matching key. The result was a dataset that included a row for each hospital visit with matched IVDRS data. There were no missing data for the variables used in our analysis (ie, all hospital visits cases have at least one primary diagnosis code assigned).

#### Inclusion Criteria

Hospital visits occurring > 7 and < 365 days from the date of death among persons who died by suicide in Illinois between January 1, 2017–December 31, 2019 were included in the final dataset. We excluded visits within seven days of death because these could have been due to the fatal injury.

### Variable Calculation

To determine the number of related ED and inpatient visits, we compared ED discharge and inpatient admit dates and counted these as related if the ED discharge date was within one day of the inpatient admit date. We calculated the inpatient length of stay by subtracting the discharge date from the admit date for inpatient visits. The number of days between last admit date and death by suicide was calculated by subtracting the death date from last admit date and then categorized using benchmarks from prior published research studies 4 as < 30 days, 30–59 days, 60–179 days, and ≥ 180 days.

We examined the distribution of suicide risk-related visit primary diagnosis *ICD-10* codes related to four risk factors: Deliberate Self Harm,^8^ Suicidal Ideation,^9^ Mental Health Disorders (not including self-harm and suicidal ideation),^10^ and Substance Use.^11^ The *ICD-10* code groupings follow those from previously published studies.^6^ See the supplemental table for a list of *ICD-10* codes included under each of the four groups. A visit may have more than one primary diagnosis code assigne; therefore, a single visit can fall into more than one of the four groupings.

We performed analyses using Python 3.10.7 (Python Software Foundation, Beaverton, OR). We determined statistically significant differences between ED and inpatient visits using chi-square tests of significance with significance set at < 0.05.

## RESULTS

### Study Sample

There were 1,186 suicide deaths recorded in the IVDRS in 2018 and 1,376 suicide deaths recorded in 2019 for a total of 2,562 suicide decedents recorded in the IVDRS with a date of death between January 1, 2018–December 31, 2019. Of the 2,562 decedents, 960 (37.4%) suicide decedents had a hospital visit > 7 and < 365 days prior to their death.

### Decedent Demographics by Visit Type

As seen in Table 1, just over two-thirds of visits were made by men. We found no statistically significant sex differences in the proportions of ED vs inpatient visits. The majority of ED and inpatient visits were made by non-Hispanic White persons, followed by non-Hispanic Black persons and persons of Hispanic descent. While differences in the proportion of visits by race were relatively small across ED and inpatient visit types, the differences were statistically significant.

### Hospital Visits

The 960 decedents had a total of 3,131 hospital visits, or 3.3 per person. Of the 3,131 visits, 2,002 (63.9%) were to the ED; slightly more than one-third (1,129, 36.1%) were inpatient visits. Overall, the number of ED visits per decedent (mean 2.1 [SD 4.1]) was higher than that for inpatient visits (1.2 [2.4]). The number of visits ranged from 0–84 to the ED and 0–40 to inpatient units. For inpatient visits, the average stay was 6.49 days (SD 8.29). The number of days per visit ranged from < 1 to 139.

Most of the 1,637 ED visits (81.8%) were independent of an ED visit as were most inpatient visits (859, 74.5%). Nearly half (441, 45.9%) of decedents had only ED visits. Less than one-fifth (166, 17.3%) had only inpatient visits. Slightly over one-third (36.8%, 353) had both ED and inpatient visits. Of those 353 decedents with both ED and inpatient visits, less than half (42.7%, 150) had an ED visit that led directly to an inpatient visit, meaning there was a temporal separation between the ED and inpatient visit, making it likely they did not occur for the same incident. A small proportion (5.1%, 161) of visits were to psychiatric hospitals. Among the psychiatric hospital visits, 49 (30.4%) were to the ED and 112 (69.5%) were inpatient.

### Admission Source

Most visits were initiated from non-healthcare facilities for both ED and inpatient. About one-fifth (20.7%, 222) of inpatient visits were transferred from healthcare facilities other than the one to which they were admitted. The differences in the distribution of ED and inpatient visit admission sources were statistically significant. For psychiatric hospitals, the pattern was different. Over half (55.5%, 60) of psychiatric hospital inpatient visits were by patients admitted as a transfer from another healthcare facility, whereas (95.9%, 47) of psychiatric hospital ED visits came from non-healthcare facilities.

### Visit Timing

We examined the timing of the last hospital visit recorded prior to death. For both ED and inpatient, the largest proportion of last visits occurred < 180 days prior to death (52.1%, 1,060, and 60.9%, 687, respectively). Almost one-sixth, (14.3%, 161) of last inpatient visits occurred < 30 days prior to death, whereas less than one-tenth (9.3%, 186) of ED visits occurred in that time range. In general, inpatient last visits were in closer proximity to death compared to ED visits. Differences in timing of last visit by setting were statistically significant (Table 3).

### Primary Diagnosis Codes

The number of primary diagnosis codes per visit ranged from 1–25. The average number of diagnosis codes per visit was 7.17. We examined only primary diagnoses codes reflective of known suicide risk factors—deliberate self-harm, suicidal ideation, mental health disorders, and substance use. Less than half (41.7%, 835) of ED visits had at least one *ICD-10* code that fell within one of these four risk groups, while the majority of inpatient visits (85.7%, 968) had a diagnosis code that fell within one or more of the four risk factor groups.[Table t2-wjem-27-345]

Table 4 indicates that the risk factor group present in the largest proportion of both ED and inpatient visits was substance use. Over three-quarters (75.1%, 848) of inpatient visits had substance use diagnosis codes while only slightly over a third (35.3%, 706) of ED visits had this designation. Mental health disorders had the lowest prevalence of any risk factor group examined for both ED and inpatient visits. The differences in the proportions of visits by primary diagnosis group and setting type were statistically significant across all diagnosis groups.

## DISCUSSION

Numerous studies have documented that most persons who die by suicide have healthcare visits in the year prior to their death, although rates vary by setting. We examined ED and inpatient hospital visits as specific forms of healthcare contacts in the year prior to death by suicide and used hospital discharge data points to evaluate the potential of these settings to reach those at risk of suicide.

In our study, just over one-third of all Illinois suicide decedents who died over a two-year period (2018–2019) had a hospital visit recorded in the year prior to their death, a significantly lower proportion than other studies found when considering all types of healthcare visits including hospital visits, primary, and specialty care.^4^,^5^ Studies focusing on primary care visits found much higher visit rates than we found for hospital visits. This suggests that while hospital settings are a vital part of the care networks used by people in the year prior to their death by suicide, there are likely other settings where a larger proportion of these individuals seek and receive care.

Overall, over half of last hospital visits occurred < 6 months prior to suicide death. Last visits closer in proximity to suicide death may present more opportunities for intervention, especially for acute problems contributing to suicide.^6^ Visits in an inpatient setting had a larger proportion of last visits occurring < 30, between 30–59, and 60–179 days prior to death compared to those in ED settings. This finding may indicate that inpatient settings have a slight advantage over ED settings in reaching those at a critical time preceding suicide.

Visits with primary diagnoses codes related to known suicide risk factors offer opportunities for suicide risk screening and, for those who screen positive, intervention delivery. Inpatient visits had a greater proportion of primary diagnoses codes related to suicide risk factors compared to ED visits, suggesting that the inpatient environment may have more potential to reach individuals at higher risk levels.

The prevalence of primary diagnosis codes for substance use among both ED and inpatient visits may bring additional challenges for delivery of suicide prevention interventions in these settings. People under the influence of substances may not be amenable to or able to participate fully in screenings.^12^ Given the higher proportion of visits related to substance use in inpatient settings, inpatient visits may offer more opportunity to reach those at risk of suicide. Furthermore, the inpatient setting may be preferable to the ED for incorporating suicide risk screening for patients with substance- and alcohol use disorders as they may be held long enough for intoxication to wear off and be more amenable to screening services, whereas in the ED these patients are released as soon as they are determined to be “clinically sober” (ie, to have reached a blood alcohol level of < 80 mg per deciliter but not be completely sober). In the context of the ED setting, this may result in a temporary risk reduction.^13^ However, as suicide risk is strongly associated with intoxication, elevated risk may reemerge upon a return to intoxication after release, creating a cycle of short-term intervention that is unable to address longer term risk. While this limitation is not unique to the ED, the shorter duration of care compared to inpatient settings may introduce additional challenges for sustained risk mitigation.

Known suicide risk factors are imperfect for predicting suicide, and many suicides occur without any risk factors present.^7^ However, this does not mean suicides without identified risk factors are not preventable. There is new and growing evidence that a sub-group of suicides may involve suicide crisis syndrome and this diagnosis may be more accurate in detecting risk for a sub-group of suicides than well-known risk factors such as histories of prior attempts and suicidal ideation.^8^ Suicide crisis syndrome is still emerging as a diagnosis; while information regarding it continues to develop we encourage exploration of this approach, as it may improve upon suicide prevention practice in healthcare.

## LIMITATIONS

The retrospective study design presents limitations including potential bias and lack of ability to control for potentially confounding variables requiring careful interpretation of findings. Furthermore, this study only included information on individuals who died by suicide; due to data agreement limitations we were unable to obtain IHDD for persons who did not die by suicide. Because the IVDRS data include only fatalities, we could not capture suicide attempts that did not result in death. Another limitation was the lack of ability to control local variation in hospital admission practices. Indications about visit content in addition to primary diagnosis code could provide additional insight into these visits, but those data were unavailable.

Analysis of all primary diagnosis codes, not just those reflecting suicide risk, could provide additional insights beyond the scope of this study, which we plan to pursue in future work. Comparisons between patients who did and did not have a hospital visit within a year of their death may add additional insight; however, those data were not available to the research team. While it would have been helpful to know whether the recorded IHDD visits included suicide risk screenings, this information was not included in the IHDD. Finally, the data for this study are from the state of Illinois, which may differ from other settings in terms of hospital visit behaviors.

## CONCLUSION

We found that the proportion of suicide decedents who visited a hospital in the year prior to death was lower than what has been reported in other studies with regard to primary care settings. However, this result does not mean that broad-based suicide screening and interventions would not be of value in these settings.^7^, ^14^ When considering the possibility of converting missed-opportunity visits to intervention opportunities, it appears that inpatient settings may offer slight advantages over the ED. Comparing ED and inpatient visits in the year prior to suicide death, we found that visits to the ED comprised the larger share of visits, but that inpatient settings had a greater proportion of visits in closer proximity to death by suicide and a greater proportion of visits associated with suicide risk factors. This suggests that in terms of expanding the reach of suicide prevention in hospital settings, EDs may be better suited to broad-based screening operations and inpatient settings to targeted efforts.

## Supplementary Information



## Figures and Tables

**Table 1:** In a cross-sectional study of Illinois hospital visits by patients in the year prior to their death by suicide, patient characteristics varied slightly by visit type (2017–2019).

	ED (%[n])	Inpatient (%[n])
*Sex*		
Male	69.1% (1,383)	69.2% (781)
Female	30.9% (619)	30.8% (348)
*Race*		
Non-Hispanic White*	71.4% (1,429)	69.2% (781)
Non-Hispanic Black*	16.3% (327)	14.5% (164)
Hispanic*	5.7% (115)	4.9% (55)
Asian non-Hispanic*	1.2% (24)	2.1% (24)
American Indian/Native Alaskan, non-Hispanic *	^	< 5
Other*	4.8% (97)	9.1% (103)

^ n < 5 suppressed due to privacy protections.

* *P* < .05.

*ED*, emergency department.

**Table 2 t2-wjem-27-345:** In a cross-sectional study of Illinois hospital visits by patients in the year prior to their death by suicide, admission sources varied by visit type, (2017–2019).

	ED % (n)	Inpatient % (n)
Non-healthcare facility*	96.0% (1,919)	68.6% (736)
Clinical physician*	1.4% (47)	4.7% (50)
Transfer from a differ facility*	^ <5	20.7% (222)
Transfer from SNF/ICF*	0.3% (6)	1.7% (18)
Transfer from another health care facility*	0.5% (9)	2.6% (28)
Court/Law enforcement*	0.8% (16)	^ <5
Information not available*	0.9% (17)	1.4% (15)

^ n < 5 suppressed due to privacy protections.

* *P* < .05.

*ED*, emergency department; ICF, intermediate care facility; *SNF*, skilled nursing facility.

**Table 3:** In a cross-sectional study of Illinois hospital visits by patients in the year prior to death by suicide, more than half of emergency department and inpatient visits occurred < 180 days prior to death (2017–2019).

	ED visits (% [n])	Inpatient visits (% [n])
< 30 days*	9.3% (186)	14.3% (161)
30–59 days*	10.8% (216)	12.9% (146)
60–179 days*	32.9% (658)	33.7% (380)
≥ 180 days*	47.1% (942)	39.1% (442)

* *P* < 0.05.

*ED*, emergency department.

**Table 4:** In a cross-sectional study of Illinois hospital visits by patients in the year prior to their death by suicide, the highest proportion of visits involving suicide risk factors were substance use-related (2017–2019).

*ICD-10* code group	ED visits (% [n])	Inpatient visits (% [n])
Deliberate self-harm*	6.8% (136)	22.2% (251)
Suicidal ideation*	8.6% (173)	29.0% (327)
Mental health disorders*	3.1% (62)	5.7% (64)
Substance use*	35.3% (706)	75.1% (848)

* *P* < 0.05.

*ED*, emergency department; *ICD-10*, International Classification of Diseases, 10^th^ Rev.
